# SAP97 Binding Partner CRIPT Promotes Dendrite Growth *In Vitro* and *In Vivo*

**DOI:** 10.1523/ENEURO.0175-17.2017

**Published:** 2017-12-06

**Authors:** Lei Zhang, Angela Marie Jablonski, Jelena Mojsilovic-Petrovic, Hua Ding, Steven Seeholzer, Ian Paterson Newton, Inke Nathke, Rachael Neve, JinBin Zhai, Yuan Shang, Mingjie Zhang, Robert Gordon Kalb

**Affiliations:** 1 Department of Pediatrics Division of Neurology, Research Institute Children’s Hospital of Philadelphia, Philadelphia, PA 19104; 2 Neuroscience Graduate Group, Department of Neuroscience, University of Pennsylvania, Philadelphia, PA 19104; 3 Proteomics Core, Research Institute, Children’s Hospital of Philadelphia, Philadelphia, PA 19104; 4 Cell and Developmental Biology School of Life Sciences, University of Dundee, Dundee DD15EH, Scotland; 5 Department of Brain and Cognitive Sciences, McGovern Institute for Brain Research at the Massachusetts Institute of Technology, Cambridge, MA 02139, USA; 6 Division of Life Science State Key Laboratory of Molecular Neuroscience, Hong Kong University of Science and Technology, Kowloon China Clear Water Bay, Hong Kong; 7 Department of Neurology Perelman School of Medicine, University of Pennsylvania, Philadelphia, PA 19104

**Keywords:** AMPA, CRIPT, dendrite, development, GluA1, motor

## Abstract

The dendritic tree is a key determinant of neuronal information processing. In the motor system, the dendritic tree of spinal cord neurons undergoes dramatic remodeling in an activity-dependent manner during early postnatal life. This leads to the proper segmental spinal cord connectivity that subserves normal locomotor behavior. One molecular system driving the establishment of dendrite architecture of mammalian motor neurons relies on AMPA receptors (AMPA-Rs) assembled with the GluA1 subunit, and this occurs in an NMDA receptor (NMDA-R)-independent manner. The dendrite growth promoting activity of GluA1-containing AMPA-Rs depends on its intracellular binding partner, SAP97, and SAP97’s PDZ3 domain. We show here that cysteine-rich interactor of PDZ3 (CRIPT) is a *bona fide* SAP97 PDZ3-domain binding partner, localizes to synapses with GluA1 and SAP97 along the dendritic tree, and is a determinant of the dendritic growth of mammalian spinal cord neurons. We further show that CRIPT has a well-conserved ortholog in the nematode, *Caenorhabditis elegans*, and animals lacking CRIPT display decreased dendrite branching of the well-studied PVD neuron *in vivo*. The lack of CRIPT leads to a selective defect in touch perception, and this is rescued by expression of wild-type (WT) human CRIPT (hCRIPT) in the nervous system. This work brings new light into the molecular machinery that drives dendritic growth during development and may prove relevant to the promotion of nervous system plasticity following insult.

## Significance Statement

Proper dendritic growth is a critical step in the development of neuronal connectivity that underlies proper neuronal communication. Much is known about how NMDA receptors (NMDA-Rs) drive neuronal development and plasticity, but less is known about how AMPA receptors (AMPA-Rs) contribute in an independent manner. While SAP97 plays a critical role in this process, the molecular mechanisms and binding partners that subserve these effects are under active exploration. Here, we show that the cysteine-rich interactor of PDZ3 (CRIPT) is a *bona fide* binding partner of SAP97 in biochemical assays and resides in dendrites in the vicinity of putative AMPAergic synapses. In knockdown experiments, we find that CRIPT is essential for SAP97-dependent dendrite growth *in vitro*. We extend these studies to an *in vivo* model and show that CRIPT is also essential for dendrite growth and mechanosensory function in *Caenorhabditis elegans*. This work links AMPA-Rs, MAGUKs, and CRIPT to essential neuronal cell biology and *C. elegans* behavior.

## Introduction

Activity-dependent development occurs throughout the neuroaxis, including the spinal cord, during early postnatal life (Inglis et al., 2002; Haas et al., 2006; Ni and Martin-Caraballo, 2010). During early postnatal life, motor neurons express a distinct set of excitatory glutamate receptor subunits and this coincides with the period of extensive dendrite growth and remodeling (Jakowec et al., 1995a,b; Stegenga and Kalb, 2001; Jeong et al., 2006). These alterations in dendritic architecture are confined to a critical period and this is subserved by the unique repertoire of glutamate receptors expressed by young spinal cord neurons (Jakowec et al., 1995a,b; Inglis et al., 2002; Jeong et al., 2006).

Activity-dependent growth of the motor neuron dendritic tree is driven by two parallel glutamate receptor mediated processes: (1) an NMDA receptor (NMDA-R)-mediated mechanism (Kalb, 1994) and (2) an NMDA-R-independent mechanism (Zhang et al., 2008). This later process depends on the activation of AMPA receptors (AMPA-Rs) assembled with the GluA1 subunit and its intracellular binding partner, synapse associated protein of 97 kDa (SAP97; Sans et al., 2001; Zhang et al., 2008; Zhou et al., 2008). Animals lacking the GluA1 subunit or SAP97 in motor neurons have a stunted dendritic tree and suffer locomotor impairments that persist throughout life (Zhang et al., 2008; Zhou et al., 2008). All of the dendrite promoting actions of GluA1 are mediated by SAP97 (Zhou et al., 2008). Trafficking of SAP97 to the cell surface is dependent on the interaction with GluA1, but GluA1 traffics to the cell surface independent of its interaction with SAP97 (Kim et al., 2005; Zhang et al., 2008). SAP97 is a postsynaptic density (PSD) scaffolding protein with several protein-protein interacting domains organized into the following structure: NH_2_ -L27-PDZ1-PDZ2-PDZ3-SH3-U5-GUK-COOH. Recent work indicates that the binding of ligand(s) to the PDZ3 domain of SAP97 is required for SAP97 to promote dendrite growth (Jablonski and Kalb, 2013; Zhang et al., 2015).

To understand the molecular mechanism by which GluA1 and SAP97 promote activity-dependent dendrite growth, we asked: (1) what proteins bind to the PDZ3 domain of SAP97? and (2) what role do such proteins play in dendritogenesis? Here, we focus on cysteine-rich interactor of PDZ3 (CRIPT), a 12-kDa protein that localizes to excitatory synapses and links proteins such as PSD protein of molecular weight 95 kDa (PSD95) to microtubules (Niethammer et al., 1998; Passafaro et al., 1999). Because PSD95 and SAP97 have a similar domain structure and can functionally complement one another in certain assays (Schlüter et al., 2006; Howard et al., 2010), we asked whether CRIPT was a SAP97 PDZ3 ligand and whether it could promote dendrite growth.

## Materials and Methods

### Antibodies

The following antibodies were used in biochemical assays: immunoprecipitation of SAP97 (Thermo Fisher Scientific, catalog #PA1-741), immunoblotting and immunoprecipitation of CRIPT (Protein Tech Group, catalog #11211-1-AP), immunoprecipitation and immunoblotting of HA-tag (BioLegend, 16B11, catalog #901512), immunoblotting of SAP97 (NeuroMab/Antibodies, catalog #73-030), immunoprecipitation and immunoblotting of the myc-tag (Cell Signaling Technology, 9B11 catalog #2276), immunoblotting GluA1 (NeuroMab/Antibodies, catalog #75-327), immunoblotting GluA2 (NeuroMab/Antibodies, catalog #75-002), immunoblotting GluA4 (Cell Signaling Technology, catalog #8070), immunoblotting NR1 (BD PharMingen, catalog #556308), immunoblotting NR2A (Alomone Labs, catalog #AGC002), immunoblotting NR2B (Alomone Labs, catalog #AGC003), and immunoblotting actin [Cell Signaling Technology, catalog #3700 (mouse) or Sigma-Aldrich, catalog #A2066 (rabbit)]. The adenomatous polyposis coli (APC) antibody used was described previously (Midgley et al., 1997). The following antibodies were used for immunocytochemistry: extracellular epitopes of GluA1 (Alomone Labs, catalog #AGC004), HA-tag [BioLegend, 16B11, catalog #901512 (mouse)], HA-tag [Santa Cruz Biotechnology, Y-11 (rabbit), catalog #SC-805], and synaptophysin (Sigma-Aldrich, catalog #SAB4502906). Secondary antibodies used included for immunocytochemistry: Alexa Fluor 633 or 568 Goat Anti-Mouse IgG (H + L; Invitrogen) and Alexa Fluor 633 or 568 Goat Anti-Rabbit IgG (H + L; Invitrogen). When primary antibody was omitted (or preimmune serum was used), staining with these secondary antibodies yielded no fluorescent signal, thus confirming the specificity of immunocytochemical signal. Secondary antibodies for Western blottings (IR DYE) comes from Li-COR.

### Mixed spinal cultures

Rat mixed spinal cord neuron cultures were prepared as previously described (Jeong et al., 2006; Zhang et al., 2015). They were maintained in glia-conditioned medium supplemented with trophic factors (Alomone Labs at a concentration of 1.0 ng/ml): human neurotrophin-3, human neurotrophin-4, human brain-derived neurotrophic factor, human cardiotrophin-1, human glial-derived neurotrophic factor, and rat ciliary neurotrophic factor. One half of the medium was replaced three times per week.

### microRNA (miRNA) RNAi design and herpes simplex virus (HSV) infection

miRNA was constructed by annealing two oligomers of 21 base pairs homologous to the CRIPT sequence together and adding BspE1 and FseI restriction sites to the 5’ and 3’ ends, respectively. Annealed oligomers were subsequently cloned into the p1006+ HSV vector using the BspEI and FseI restriction sites. miRNA was designed against the rat CRIPT sequence corresponding to the ORF: 5’-CTCCACTTGCAGAATTT-3’. We generated an RNAi-resistant CRIPT cDNA containing three silent mutations in the miRNA target sequence of CRIPT by using the QuikChange Site-Directed Mutagenesis kit using manufacturer’s protocol (Agilent). The miRNA target sequence was mutated to: 5’-CTCTACCTGTAGAATTT-3’. Mutated nucleotides are underlined. The p1006+ vector was subsequently used for HSV production as previously reported (Neve et al., 1997) . The titer of virus used in these experiments was 3–5 × 10^7^ pfu/ml.

### Heterologous cells and transfection

For all experiment except those involving APC, HEK293 cells were maintained in 5% CO_2_ at 37°C in DMEM (Invitrogen) supplemented with 10% fetal bovine serum (FBS; Sigma), 1% penicillin/streptomycin (Pen/Strep; Sigma). Cells were transfected (Lipofectamine 2000; Invitrogen) per manufacturer’s protocol when they were ∼75% confluent and were maintained in DMEM with 10% FBS until lysed 48 h after transfection.

For experiments involving APC, HEK293 cells were maintained at 37°C in 5% CO_2_ in DMEM (Life Technologies), supplemented with 10% FBS (GE Health Care Life Sciences), 1% Pen/Strep (Life Technologies), and nonessential amino acids (1:100; Life Technologies). Cells were transfected using FuGENE 6 (Promega), according to the manufacturer's instructions.

### Immunoprecipitation and Western blotting

For immunoprecipitation experiments not involving APC, lysates were made in 1% NP-40 lysis buffer (25 mM Tris-HCl, pH 7.4, 150 mM NaCl, 1 mM EDTA, 1% NP-40, 5% glycerol; 150 μl per 60-mm dish) supplemented with fresh protease and phosphtase inhibitor cocktail (Sigma). After two washes in ice-cold 1× PBS, cells were lysed in lysis buffer, sonicated (20% strength, 10 s), and centrifuged at 13,000 × *g* for 10 min (4°C) to remove cellular debris. Five percent of the lysate was saved for input, and the remaining lysate was used for immunoprecipitation. For immunoprecipitation, DynaBeads Protein G (Invitrogen) were precleared in PBS/1% Tween and were bound to the antibodies (4 μg of antibody; 30 min at room temperature, RT). Lysate was added, incubated for 1 h at RT, and the lysate and bead mixture was washed three times in lysis buffer. Proteins were then boiled in 1% BME and 1× SDS loading buffer (40 µl total). Proteins were immunoblotted following standard Western blotting technique using 4-12% Bis-Tris gels (Invitrogen) in MES-SDS running buffer (Invitrogen). Bio-Rad SDS-PAGE standards Low Range (catalog #161-0305) molecular weight markers were used. Gels were transferred onto nitrocellulose (Bio-Rad) at 100V for 1 h at 4°C and blocked in 5% milk in TBST buffer (50 mM Tris-HCl, pH 7.4, 150 mM NaCl, 0.1% Tween 20). Primary antibodies were incubated in blocking buffer as indicated overnight at 4°C. Secondary anti-rabbit and anti-mouse Alexa Fluor 800/680-conjugated antibodies (Life Technologies) were diluted 1:10,000 in 5% milk in TBST buffer and binding was detected using the Odyssey system (LiCor) Infrared Imaging System without saturating the protein bands. The intensity values were normalized against the actin loading control. Quantitative data were the average of no fewer than three separate and independent experiments.

For immunoprecipitation experiments involving APC, lysates were made using MEBC lysis buffer (50 mM Tris, pH 7.5, 100 mM NaCl, 5 mM Na-EDTA, 5 mM Na-EGTA, 40 mM β-glycerophosphate, and 0.5% NP-40) containing a cocktail of protease inhibitors: leupeptin, pepstatin A, chymostatin (each 10 μg/ml), 1 mM NaVO_4_, and 10 mM NaF. Cells were scraped and the mixture was spun at 14,000 rpm for 20 min at 4°C. Supernatants were removed and immediately frozen using liquid nitrogen. To measure the binding of endogenous APC to either wild-type (WT) or mutant SAP97, 10 μg of polyclonal anti-SAP97 antibody (Fisher Scientific) was prebound to 100 μl of 50% protein-A-Sepharose bead slurry (Sigma) by gently agitating the mixture at 4°C for 16 h. After washing the beads twice with MEBC, 0.5 mg of lysate from cells transfected with WT or mutant SAP97 was added and gently mixed for 16 h at 4°C. The beads were washed three times with cold MEBC buffer before they were resuspended in 20 μl SDS-PAGE loading buffer and heated to 70°C. Samples (without the beads) were subjected to SDS-PAGE using 4-12% gels (Life Technologies) in MOPS-SDS running buffer (Life Technologies). Proteins were transferred to GE Healthcare Protran nitrocellulose membrane (0.1 μm; GE Health Care Life Sciences). Blots were probed with crude serum from rabbits immunized with N-terminal portion of APC (Midgley et al., 1997) diluted at a concentration of 1:1000 in blocking solution (Tris-buffered saline, 5% nonfat milk, 1% goat serum, and 0.02% Triton X-100) and an anti-SAP97 antibody (Fisher Scientific) diluted 1:1000. Secondary anti-rabbit and anti-mouse Alexa Fluor 800/680-conjugated antibodies (Life Technologies) were diluted 1:10,000 and binding was detected with Odyssey (LiCor).

### Surface plasmon resonance (SPR) sample preparation

The PDZ3 domain of SAP97 was amplified by PCR and the PCR product was ligated into the pGEX vector (GE) and transformed into BL21 strain. Correctness of the construct was confirmed by sequencing. GST-PDZ3 fusion protein expression was induced with isopropyl-1-thio-β-d-galactopyranoside (IPTG) for 3 h. The bacteria were harvested by centrifugation, resuspended in ice-cold lysis buffer (50 mM Tris, 50 mM NaCl, 5 mM EDTA, 1 μg/ml leupeptin, 1 μg/ml pepstatin, 0.15 mM PMSF, and 1 mM 2-mercaptoethanol), and lysed by sonication on ice. The bacterial lysates were cleared by centrifugation, and supernatant was collected and incubated with glutathione Sepharose 4B matrix. After extensive washes with lysis buffer, glutathione Sepharose 4B resin was incubated with freshly made 10 mM glutathione buffer (50 mM Tris and 10 mM reduced glutathione, pH 8.0) to elute the GST fusion protein. Purified GST-PDZ3 fusion protein was further dialyzed to remove glutathione before plasmon resonance studies.

### SPR measurements and affinity analysis of sensorgrams

Association and dissociations reactions of peptides CHRK1 (C-terminal amino acids of CRIPT) or CHRK2 (C-terminal amino acids of CRIPT V101A; as analytes) and recombinant GST fusion protein GST-PDZ3 and GST (as ligands) were performed using a Biacore3000 instrument. Anti-GST antibody was immobilized on flow cell 3 and 4 according to the manufacturer’s instructions. GST and GST-PDZ3 were then captured on flow cell 3 and flow cell 4 separately, where GST served for on-line reference subtraction (values shown in data subtract any signal from this negative control). Both the analytes and the ligands were dissolved in the HBS running buffer (0.01 M HEPES, 0.15 M NaCl, 3 mM EDTA, and 0.005% v/v Surfactant P20), and their concentrations were determined at UV 280 nm using extinction coefficients calculated from the amino acid composition. The ligands were bound as densities between 1200 and 1700 RU. The analytes of CHRK1 and CHRK2 in 45 µl volume each with various concentrations (62.5 nM, 250 nM, 4 µM, 8 µM, 10.24 µM, and 12.8 µM) were injected over both GST and GST-PDZ3 surfaces at flow rate of 30 µl/min. Dissociation time was monitored for 3 min. A 40-s pulse of glycine-HCl, pH 2.2, was applied to regenerate the GST surface. For GST-PDZ3 surface regeneration, an additional 40-s pulse of glycine-HCl was applied. Analysis of steady-state affinity was performed using BIAevaluation software selecting reference-subtracted curves with a 1:1 interaction model. Affinity constant *K*_d_ was derived from Req versus C (response unit at equilibrium vs peptide concentration) plot with the steady-state affinity model fit. Data for GST capture was subtracted from GST-PDZ3 values.

### Neuronal transfection and neuron tracings

At 5 d *in vitro* (DIV), mixed spinal cultures were transfected (Lipofectamine 2000; Invitrogen) with the overexpression plasmid or miRNA being tested in a 3:1 ratio with GFP to ensure all GFP-positive cells also expressed the desired construct. Five days after transfection, cells were fixed in 4% paraformaldehyde and immunostained with rabbit anti GFP antibody and secondary antibody. After immunostaining, the coverslip with the cells was mounted on the slide and viewed using fluorescent microscopy. Neuronal tracings were performed with the Neurolucida program (MicroBrightField) and analyzed with the Neuroexplorer program (MicroBrightField). As previously described (Zhang et al., 2008), the minimum inclusion criteria for this study were: dendrites radially distributed (>180°) and no more than one primary dendrite is truncated (less than three times the cell body diameter). In addition, only processes ≥4 μm were included in our analysis since this distinguishes true branches from dendritic filopodia (Zhang et al., 2015). Quantitative descriptors of the dendrites were as follows (Zhang et al., 2015): 1° dendrites: the number of primary dendrites leaving the cell body; branches, #: the number of dendritic bifurcations; Σlength: sum of the linear length of all dendrites from a single cell; average dendrite: the sum of the lengths of all dendritic shafts from a single neuron divided by the number of primary dendrites; and longest dendrite: the length of the longest dendrite from the cell body to the most distal tip. Spinal cord cultures were created over a six-year period for these experiments and all the anatomic drawings were performed by a single operator (L. Zhang) blinded to experimental group. All drawings of neurons within a specific experiment (three groups in Fig. 5, 6) were done contemporaneously. Since specific experiments were performed over 6 years, absolute values obtained for one specific experiment cannot be directly compared with the absolute values obtained for a second specific experiment. Sources of variation include differences in batches of serum, Dams from different venders, etc.

### Immunocytochemical localization of CRIPT in mixed spinal cord cultures

We found none of the commercially available antibodies to CRIPT or SAP97 were suitable for immunocytochemical studies (e.g., Abcam; ProteinTech Group; Santa Cruz Biotechnology, NeuroMab). We devised an alternative strategy for visualizing CRIPT in neurons. We cotransfected neurons with a miRNA designed to knockdown endogenous CRIPT alongside an RNAi-resistant version of WT CRIPT containing a hemagglutinin (HA) tag on the amino terminus. When these plasmids were cotransfected with a GFP reporter and stained for HA, we visualized punctate HA immunoreactivity in the cell body and dendrites of transfected neurons. The molar ratio of the transfected plasmids (e.g., GFP:CRIPTmiRNA:RNAi resistant HA-CRIPT was 1:5:2) to ensure that HA staining was restricted to neurons with a high likelihood of concomitant knockdown of endogenous CRIPT. Both mouse anti-HA (BioLegend) and rabbit anti-HA (Santa Cruz Biotechnology) worked well in this application. In one set of experiments, we used a mammalian expression vector engineered to express GFP-SAP97 in localization studies.

### Membrane protein preparations

Cells were washed twice using ice-cold PBS and suspended in hypotonic lysis buffer (10 mM KCl, 1.5 mM MgCl_2_, and 10 mM Tris-Cl, pH 7.4, and 1.5 ml/60 mm plate). After incubation on ice for 10 min, cell lysis was finalized using a homogenizer (Dounce). The cell lysate was centrifuged at 2000 × *g* for 2 min (4°C) to remove nuclei and cellular debris and the supernatant was centrifuged a second time at 100,000 × *g* for 30 min at 4°C to pellet cell membranes. The supernatant was saved as the “cytoplasmic” fraction and the pellet (“membrane” fraction) was resuspended in RIPA buffer (50 mM Tris-HCl, pH 7.5, 150 mM NaCl, 1 mM EDTA, 1 mM EGTA, 1% NP-40, and 1% sodium deoxycholate) supplemented fresh with protease and phosphatase inhibitor cocktail (Sigma). Lysates were boiled for 5 min in 1% BME (Bio-Rad) and 1× SDS-loading buffer before standard Western blotting technique as described earlier using 4-12% Bis-Tris gels (Invitrogen) in MES-SDS buffer (Invitrogen).

### Yeast-two-hybrid (Y2H)

The PDZ3 domain of SAP97 flanked by five amino acids on the N and C termini was amplified by PCR and cloned into the yeast bait phagemid pBD-GAL4. Full-length (FL) CRIPT was engineered into the yeast prey phagemid pAD-UAS. YRG-2 yeasts were transformed (Clontech) according to the manufacturer’s instructions with phagemids or appropriate empty vector controls and grown under restrictive conditions (SD media lacking leucine, tryptophan, and/or histidine) and single colonies were subsequently streaked onto restrictive plates. The plasmid, pGBT9, was used as a positive control.

### CRIPT and SAP97 structural modeling

The SAP97 PDZ3 model was built in SWISS-MODEL on-line server (http://swissmodel.expasy.org/). PSD95 PDZ3/CRIPT (PDB id:1BE9) was selected as the template in the modeling process. For the SAP97/PDZ3/CRIPT complex, CRIPT peptide was modeled from PSD95 PDZ3/CRIPT complex (PDB id:1BE9). Then, both the complex structure was further adjusted manually to take the N-terminal upstream residues of CRIPT into account. Finally, the structure model was submitted to YASARA Energy Minimization server (http://www.yasara.org/minimizationserver.htm) to achieve the final SAP97/CRIPT complex model.

### Caenorhabditis elegans maintenance

All *C. elegans* strains were maintained using standard methodology (Brenner, 1974). Animals were maintained at 20°C on nematode growth medium (NGM) agar plates with live OP50 bacteria. A worm strain containing a deletion within the *C. elegans* ortholog of CRIPT, *tm430*, was obtained from National BioResource Project in Japan and backcrossed three generations to ancestral N2 before being crossed into the P_PVD_::GFP line. We also used the *mec-4* (*u253*) and *mec-3* (*e1338*) strains, which were obtained from the Caenorhabditis Genetics Center (CGC). The *mec-4* (*u253*) strain was backcrossed three generations to ancestral N2 before being analyzed and crossed into the *tm430* strain.

### C. elegans rescue cloning and lines

Human CRIPT (hCRIPT) cDNA was amplified using PCR. Gateway cloning (Invitrogen) was employed and the BP reaction was used to construct the pDonR221-hCRIPT (pEntry::hCRIPT) construct. The LR reaction was then performed using the following constructs for the 1st, 2nd, and 3rd positions, respectively: unc-119 promoter, pEntry::hCRIPT, and the unc-54 3’UTR. The LR reaction was performed to insert this cassette into the pCFJ150 destination plasmid for *C. elegans* expression. Sanger sequencing was used to confirm the correctness of the final construct, P_unc-119_::hCRIPT::unc-54 3’UTR (P_neuron_::hCRIPT) enabling the expression of hCRIPT in the *C. elegans* nervous system. To create transgenic extrachromasomal arrays, P_neuron_::hCRIPT was injected into the gonads of the indicated strains (at 1 d after the fourth larval stage, L4) at a final concentration of 50 ng/μl along with a coinjection marker, pCFJ150 (P_myo-2_::mCherry) at a final concentration of 5 ng/μl. The final DNA concentration of the injection mix was 100 ng/μl; a sonicated LacZ plasmid was used to arrive at the proper final DNA concentration as needed. At least three independent transgenic lines were created for each final strain, and all were tested in corresponding assays.

### C. elegans imaging

All lines were synchronized before visualizing GFP-labeled PVD neurons. Briefly, young adult (1 d after L4) hermaphrodites were allowed to lay eggs for 4-6 h. At the L4 stage, noted by the presence of a clear vulva, animals were moved to a separate plate and imaged one day later. Animals were immobilized using levamisole (10 mM; Sigma) on fresh 4% agar pads and imaged using confocal microscopy. Z-stacks were obtained and merged, and was analyzed by manually counting the number of dendrites present on the PVD neuron dendritic tree (Smith et al., 2010, 2013) from the cell body to the posterior end of the animal. The number of primary, secondary, and tertiary dendrites was determined as previously described (Liu and Shen, 2011). The analyst was blind to experimental group during image acquisition and analysis.

### C. elegans touch assay

In the touch assay, the midsection of the body near the vulva was gently prodded with a platinum wire, as previously described (Way and Chalfie, 1989). A positive response was scored as movements of at least one body length in response to the touch stimulus, although the speed of the animal can vary in different mutant backgrounds.

### Statistics

Statistical analysis was performed using GraphPad Prism version 6.00 for Windows, GraphPad Software. Unpaired *t* test was used for all two-group comparisons. For data more than three groups, a one-way ANOVA with Dunnett’s multiple comparisons test was used. For analysis of the neuronal tracings, a Shapiro–Wilk test was run to test normality of the data (SPSS). If the data were normally distributed, the data were analyzed with one-way ANOVA. *Post hoc* analysis was performed with Tukey’s test. If the data did not distribute normally, a nonparametric Kruskal-Wallis *H* test (e.g., one-way ANOVA on ranks) was used to analyze data. *Post hoc* analysis was performed with Dunnett’s test. The threshold for significance was set at *p* < 0.05 for all tests. Data are presented as mean ± SEM.

## Results

### CRIPT interacts with SAP97’s PDZ3 domain

**Figure 1.**
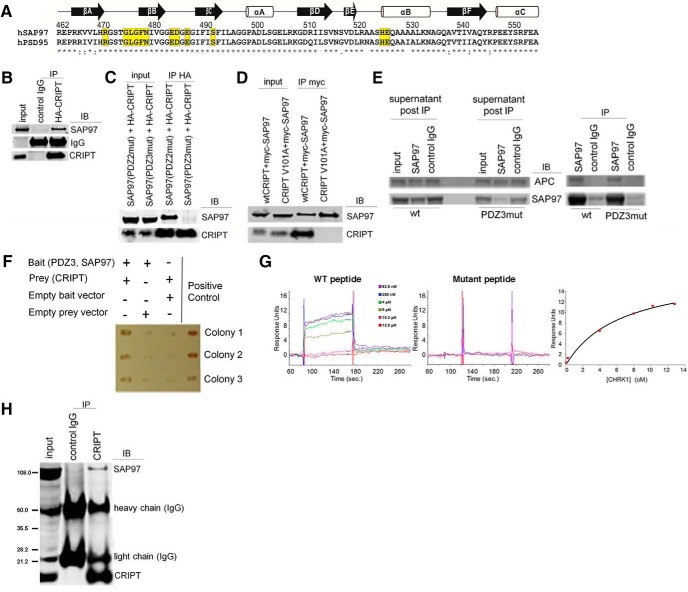
CRIPT is *bona fide* PDZ3 and SAP97 interacting protein. ***A***, Amino acid sequence alignment of the PDZ3 domains of human SAP97 and human PSD95; there is 86% identity. Residues highlighted in yellow indicate amino acids directly involved in binding the C terminus of CRIPT. Arrows and cylinders above the amino acid sequence indicate secondary structure of the PDZ domain (i.e., βA means beta strand A, αA means alpha helix A, etc.). ***B***, FL myc-tagged SAP97 coimmunoprecipitates with FL HA-tagged CRIPT using the anti-HA antibody when they are overexpressed in HEK293 cells. Neither protein immunoprecipitates with a negative control IgG. ***C***, Overexpressed HA-tagged CRIPT coimmunoprecipitates with myc-tagged SAP97 containing a mutation in the PDZ2 domain (K323A, K326A), but mutation of the PDZ3 domain (H469A, R470A) disrupts this interaction. ***D***, Overexpression of myc-tagged FL SAP97 and FL WT CRIPT or FL CRIPT V101A in HEK293 cells and immunoprecipitation SAP97 using myc IgG. Mutating the PDZ3-interaction motif of CRIPT (CRIPT V101A) abolishes the interaction between SAP97 and CRIPT. ***E***, anti-SAP97 (but not a control IgG) immunoprecipitates WT or PDZ3 mutant version of SAP97 equivalently from cell lysates, as revealed by assay for supernatant after IP. FL SAP97 interacts with FL APC protein in HEK293 cells, but this interaction does not rely on a ligand-binding PDZ3 domain in SAP97. ***F***, FL CRIPT interacts with the PDZ3 domain of SAP97 in a Y2H assay. ***G***, SPR shows a specific and saturable interaction between the 18 C-terminal amino acids of CRIPT and the PDZ3 domain of SAP97 (*K*_d_ of 6.57 μM), but no interaction when the CRIPT V101A mutation is present. Values shown were subtracted by a negative control GST peptide. ***H***, Endogenous CRIPT and endogenous SAP97 coimmunoprecipitate in lysates prepared from DIV14 mixed spinal cultures. Immunoblotting of endogenous SAP97 and CRIPT proteins in input lysate and immunoprecipitations using a negative control IgG and CRIPT IgG. Blot was incubated with SAP97 and CRIPT antibody simultaneously.

Recent work has revealed that the third PDZ domain of SAP97 is critical for the prodendrite-growth activity of SAP97 (Zhang et al., 2015). Identification of the endogenous SAP97 PDZ3 domain ligand(s) responsible for this biological activity is problematic because the list of candidates contains ∼90 potential binding partners (see “interactions” at http://www.ncbi.nlm.nih.gov/gene/25252). In an attempt to focus the search, we noted that there is 86% sequence homology between the PDZ3 domain of SAP97 and the PDZ3 domain of PSD95 (Fig. 1*A*). Since CRIPT binds to the PDZ3 domain of PSD95 (Niethammer et al., 1998), we tested CRIPT binding to the PDZ3 domain of SAP97. We used a heterologous expression system in HEK293 cells and established that FL HA-tagged CRIPT (HA-CRIPT) and FL myc-tagged SAP97 (myc-SAP97) coimmunoprecipitated (Fig. 1*B*). Anti-HA IgG, but not a control IgG, successfully coimmunoprecipitates HA-CRIPT and myc-SAP97. To determine whether the PDZ3 domain of SAP97 mediates this interaction, we attempted to coimmunoprecipitate HA-CRIPT with versions of FL SAP97 containing ligand binding-disabling mutations in either the PDZ2 (K323A, K326A) or PDZ3 (H469A, R470A) domain. We found that mutations in the PDZ3 domain, but not in the PDZ2 domain, completely abolished binding of CRIPT to SAP97 (Fig. 1*C*). Next, we attempted to coimmunoprecipitate FL myc-SAP97 with two versions of CRIPT: (1) a WT FL CRIPT and (2) a version of FL CRIPT harboring a point mutation in the C-terminal valine (the PDZ3-binding motif of CRIPT, -QTSV). This mutation was previously shown to disrupt the binding of CRIPT to PSD95 (referred to as CRIPT V101A), but not CRIPT’s binding to microtubules (Passafaro et al., 1999). We found that the V101A mutation prevented CRIPT’s binding to SAP97 (Fig. 1*D*). As a specificity control, we asked whether another SAP97-binding partner, APC, also showed differential binding. In contrast to CRIPT, APC was pulled down equivalently by WT SAP97 and PDZ3 mutant SAP97 (Fig. 1*E*). Taken together, these data suggest that CRIPT specifically interacts with the third PDZ domain of SAP97, and this interaction relies on the canonical PDZ-interaction motif of CRIPT at its C terminus.

We complemented these observations with two further approaches. First, in a Y2H assay, we found that FL CRIPT interacts with the PDZ3 domain of SAP97. FL CRIPT neither interacted with an empty bait vector, nor did the SAP97 PDZ3 domain interact with an empty prey vector (Fig. 1*F*). Second, SPR studies were performed with immobilized GST-PDZ3 or GST and peptides corresponding to the C-terminal 18 amino acids of CRIPT. We found that WT CRIPT peptide bound to the PDZ3 domain of SAP97 with a *K*_d_ of 6.57 μM, while V101A mutant showed no saturable binding to the PDZ3 domain of SAP97 (Fig. 1*G*). Prior work suggests that ligand binding to PDZ domains can vary substantially as a function of: (1) method (i.e., SPR or fluorescence polarization; Hung and Sheng, 2002) and (2) the length, in amino acids, of the peptide ligand (i.e., longer peptides displaying lower *K*_d_). Niethammer et al. (1998) showed that a peptide corresponding to the C-terminal nine amino acids of CRIPT binds to the PSD95 PDZ3 with a *K*_d_ = ∼1 µM. These results cannot be directly compared with our observations, because Niethammer et al. (1998) used fluorescence polarization as opposed to SPR. Nonetheless, our observations are within the range of reported affinities for ligand binding to isolated PDZ3 (Irie et al., 1997) and as such are consistent with a direct interaction between the PDZ3 domain of SAP97 and the C terminus of CRIPT.

To study a more physiologic setting, we asked whether CRIPT and SAP97 coimmunoprecipitated using lysates prepared from mixed spinal cord cultures. Anti-CRIPT antibody, but not a control IgG, was able to immunoprecipitate CRIPT and SAP97 together (Fig. 1*H*). Immunoreactivity at ∼50 and ∼25 kDa in the “input” lane may represent a minor amount of spill over from the adjacent “control IgG” lane. As expected, prominent immunoglobulin heavy and light chain bands are present in the immunoprecipitated material, as seen in the control IgG and the anti-CRIPT IgG lanes. This experiment confirms that endogenous CRIPT and SAP97 physically interact within a nervous system setting.

**Figure 2.**
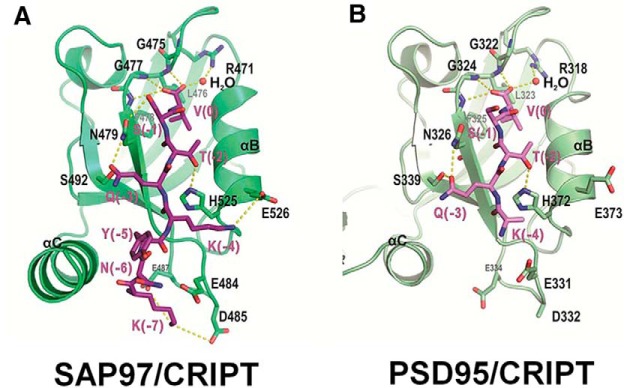
Structural model of SAP97 PDZ3 in complex with the CRIPT C-terminal tail. ***A***, The SAP97 PDZ3/CRIPT peptide complex structure modeled using the experimentally derived PSD95 PDZ3/CRIPT complex structure (PDB id:1BE9) depicted in ***B***. In these drawings, the CRIPT peptide is depicted in purple.

The above described biochemical studies make the case for a direct physical interaction between CRIPT and PDZ3 of SAP97. However, all biochemical studies come with caveats, and it remains possible that the interaction between CRIPT and PDZ3 of SAP97 (Fig. 1*B–H*) is indirect. To gain further insight into this potential binding, we attempted to model this interaction at the atomic level. We began by refining the deposited PSD95 PDZ3/CRIPT interaction and then modeled the SAP97 PDZ3/CRIPT complex based on the refined PSD-interacting features (Fig. 2). The main residues directly involved in CRIPT binding to PDZ3 of PSD95 and SAP97 are conserved (Fig. 1*A*, yellow). The C-terminal three residues of CRIPT that bind to PDZ3 of PSD95 and SAP97 are identical. In the PSD95/CRIPT structure, the side chains of D332 in PSD95 and K(-4) in CRIPT are not defined in the original crystal structure. In our modeled structure, K(-4) of CRIPT forms a salt-bridge with D332 of SAP97 PDZ3. Additionally, K(-7) of CRIPT forms a pair of salt-bridges with D485 and E487 from the beta strand B/alpha helix C (βB/αC-loop) of SAP97 PDZ3. Finally, Y(-5) of CRIPT interacts with the hydrophobic residues in the C-terminal α-helix extension of SAP97. These additional charge-charge, hydrogen bonding, and hydrophobic interactions between the N-terminal sequences of the CRIPT peptide and the αB/αC-loop of PDZ3, in addition to the canonical PDZ-binding motif and PDZ3 interactions, could enhance both the binding affinity and specificity between SAP97 and CRIPT. This new *in silico* analysis provides a plausible physical means by which CRIPT could directly interact with SAP97.

**Figure 3.**
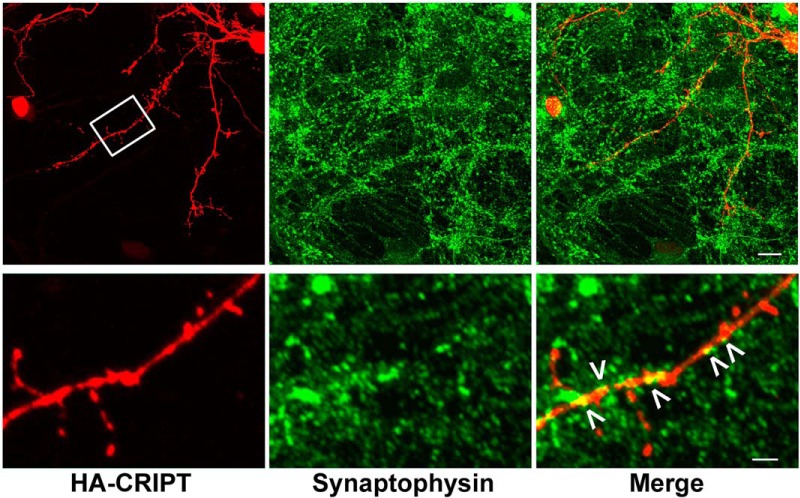
CRIPT partially colocalizes synaptophysin containing synapses. Mixed spinal cord cultures at DIV21 were immunocytochemically analyzed. The first row of panels shows immunocytochemical staining for HA-CRIPT (red), synaptophysin (green), and both together in the merge image. HA-CRIPT immunoreactivity is seen within the soma and throughout the dendritic tree. This localization data matches what was previously reported (Niethammer et al., 1998b). Scale bar: 20 μm. Beneath each of the lower power images are higher magnification images of the area outlined in a white box. In the high-power HA-CRIPT panel, immunocytochemically positive material is seen in various morphologies within the dendritic tree including small or large round puncta and elongated dendritic shaft entities. HA-CRIPT appears inhomogenously within dendritic outgrowths that may represent spines or filopodia. In the merge image, areas of overlap (HA-CRIPT + synaptophysin) are yellow and are highlighted with >. Scale bar: 5.0 μm. These are likely to represent synapses.

**Figure 4.**
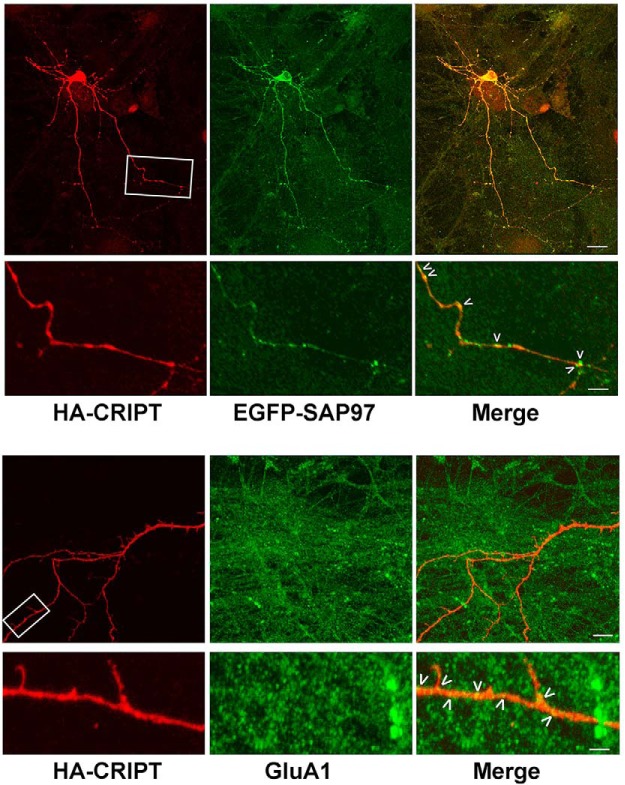
CRIPT partially colocalizes GluA1-positive and SAP97-positive puncta on dendrites. Mixed spinal cord cultures at DIV21 were immunocytochemically analyzed. The upper set of panels display images of HA-CRIPT and EGFP-SAP97, and the lower set of panels display images of HA-CRIPT and GluA1. For the HA-CRIPT/EGFP-SAP97 panels, the first row of panels shows immunocytochemical staining for HA-CRIPT (red), EGFP-SAP97 (green), and merge. As in Figure 3, HA-CRIPT immunoreactivity is seen within the soma and throughout the dendritic tree. EGFP-SAP97 is similarly distributed but appears more punctate. In the merge image, extensive colocalization is seen (yellow) both in the soma and the dendritic tree. Scale bar: 30 μm. Beneath each of the lower power images are higher magnification images of the area outlined in a white box. In the high-power HA-CRIPT panel, immunocytochemically positive material is again seen in various morphologies within the dendritic tree, including small or large round puncta and elongated dendritic shaft entities. HA-CRIPT appears inhomogenously within dendritic outgrowths that may represent spines or filopodia. EGFP-SAP97 is clearly more punctate than HA-CRIPT and in the merge image areas of colocalization are seen (yellow, denoted with >). EGFP-SAP97 appears enriched at along the edges of HA-CRIPT immunoreactivity. Scale bar: 10 μm. For the HA-CRIPT/GluA1 panels, the first row of panels shows immunocytochemical staining for HA-CRIPT (red), GluA1 (green), and merge. Again, HA-CRIPT immunoreactivity is seen within the soma and throughout the dendritic tree. GluA1 is seen exclusively as puncta. In the merge image, colocalization is seen (yellow) in the dendritic tree. Scale bar: 20 μm. Beneath each of the lower power images are higher magnification images of the area outlined in a white box. In the high-power HA-CRIPT panel, immunocytochemically positive material is again seen in various morphologies within the dendritic tree including small or large round puncta and elongated dendritic shaft entities. HA-CRIPT appears inhomogenously within dendritic outgrowths that may represent spines or filopodia. GluA1 is exclusively punctate and in the merge image areas of colocalization are seen (yellow, denoted with >). GluA1, like EGFP-SAP97, appears enriched at along the edges of HA-CRIPT immunoreactivity. Scale bar: 4.0 μm.

**Figure 5.**
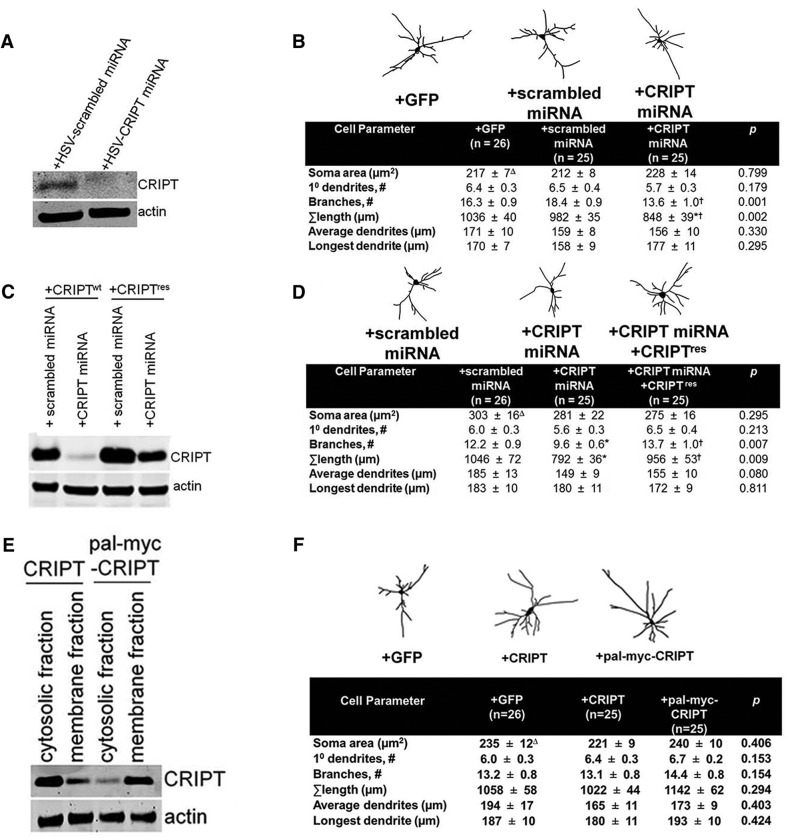
CRIPT is necessary, but not sufficient, for the dendritic growth of spinal cord neurons. Mixed spinal cord cultures were used for biochemistry and morphometry. ***A***, DIV14 cultures were infected with HSV engineered to express a miRNA that targets CRIPT or a scrambled sequence control. Lysates were probed for CRIPT and the miRNA to CRIPT reduces the abundance of CRIPT by Western blotting compared to a scrambled miRNA. ***B***, Knockdown of CRIPT results in a significant decrease in a decrease in the number of branches and total dendritic length of the dendritic tree. No other dendritic parameter was different between groups; *differs from +GFP group, *p* < 0.05; †differs from +scrambled miRNA group, *p* < 0.05. ***C***, HEK 293 cells were transfected with plasmids engineered to express miRNA targeted to CRIPT (or scrambled sequence control) and WT-CRIPT (CRIPT^WT^) or a miRNA-resistant version of CRIPT (CRIPT^res^). When lysates were probed for CRIPT, we saw reduced abundance of CRIPT^WT^ but not the CRIPT^res^ by the miRNA targeted to CRIPT. ***D***, Cotransfection of RNAi-resistant CRIPT is sufficient to prevent decrease in the number of branches and total length of the dendritic tree by CRIPT miRNA; *differs from +GFP group, *p* < 0.05; †differs from +CRIPT miRNA group, *p* < 0.05. CRIPT was modified with a sequence from paralemin-1 to allow for palmitoylation was generated. ***E***, HEK293 cells were transfected a plasmid engineered to express CRIPT^WT^ or palmitoylated CRIPT, and cells were lysed and fractionated to enrich for the cytosol or membrane fractions. Western blottings for CRIPT show that CRIPT^WT^ is predominantly in the cytosolic fraction and palmitoylated CRIPT is predominantly in the membrane fraction. ***F***, Overexpression of WT CRIPT or pal-myc CRIPT has no effect on the size or complexity of the dendritic tree compared with GFP only expressing neurons.

Previous localization work (at the light and electron microscopic levels) places CRIPT in the PSD ∼35 nm from the inner leaflet of the plasma membrane and hippocampal cultures, CRIPT colocalizes with PSD95 at excitatory synapses (Niethammer et al., 1998). In light of the known physical interaction between GluA1 and SAP97 (Sans et al., 2001; Zhou et al., 2008; Zhang et al., 2015), we explored the localization of these proteins and their relationship to synapses using immunocytochemical tools in mixed spinal cord cultures. The antisera generated to CRIPT by the Sheng lab (Niethammer et al., 1998) is no longer available and despite investigating a variety of fixation protocols, we found that none of the commercially available antibodies to CRIPT were suitable for immunocytochemical imaging of endogenous CRIPT. To overcome this difficulty, we knocked down endogenous CRIPT and coexpressed an RNAi resistant HA-tagged CRIPT (see Materials and Methods). CRIPT immunoreactivity was seen in the cell soma and throughout the dendritic tree (Figs. 3, 4), as previously reported by Niethammer et al. (1998); axons appear devoid of CRIPT immunoreactivity. This pattern of immunoreactivity is very similar to that described by Niethammer et al. (1998; Fig. 5*B*,*E*,*G*).

Niethammer et al. (1998) describe CRIPT localization to synapses on dendritic spines. In our spinal cord neuron cultures we do not typically see dendritic spines (Zhang et al., 2015), yet we find that CRIPT is juxtaposed to (and partially overlaps with) synaptophysin puncta on the shafts of dendrites (Fig. 3). Niethammer et al. (1998) describe the localization of CRIPT with PSD95, and we too find that CRIPT is juxtaposed to (and partially overlaps with) SAP97 as well as GluA1 (Fig. 4).

It is important to note, however that the focal accumulations of CRIPT that we describe here (Figs. 3, 4) and their partial colocalization with SAP97 and GluA exist within a background of diffuse CRIPT immunoreactivity within dendrites. We cannot rule out the possibility that some of the immunocytochemical signal is a result of the knockdown and replacement strategy we employ and thus only approximates the distribution of the native protein *in situ*. Given these caveats, our results suggest that a portion of dendritic shaft CRIPT apparently localizes to synapses along with components of AMPA-R complexes such as GluA1 and SAP97. This would be consistent with the immunogold electron microscopic localization studies of Niethammer et al. (1998). The precise relationship between these complexes and those defined by Niethammer et al. (1998; e.g., composed of GRIN2B, PSD95, and CRIPT) will require further inquiry. Super-resolution microscopy, array tomography (Micheva et al., 2010), and the proximity ligation *in situ* assay (Söderberg et al., 2006) are imaging technologies particularly well suited for inquiry into the fundamental relationship between these proteins.

### CRIPT is necessary for proper dendrite growth

To identify the functions of CRIPT, we began by using RNA interference (RNAi) to knockdown CRIPT in mixed spinal cord cultures. We targeted a miRNA to the rat ortholog of CRIPT, engineered it into a HSV amplicon vector, and infected DIV14 mixed spinal cord cultures with the virus and probed for CRIPT. We determined that the CRIPT miRNA, but not a miRNA to a scrambled sequence (control), effectively knocked down CRIPT in neurons *in vitro* (Fig. 5*A*). Using this tool, we knocked down CRIPT in spinal cord neurons and quantitatively analyzed dendritic architecture. To do this, we compared the dendritic tree of three experimental groups: (1) GFP alone, (2) GFP + the scrambled miRNA, and (3) GFP + active CRIPT miRNA by making camera lucida drawings of the dendritic trees in each condition. To assess the effect of CRIPT knockdown on dendritic morphology, we characterized a number of architectural features as described in Materials and Methods (Zhang et al., 2008; Zhou et al., 2008; Zhang et al., 2015). Group differences were found in branch number (Kruskal-Wallis *H* test, *H*_(2,73)_ = 14.152, *p* = 0.001) and total dendrite size (ANOVA, *F*_(2,73)_ = 6.893, *p* = 0.002). We found that knockdown of CRIPT led to a ∼30% reduction in the number of dendritic branch points compared to GFP alone (*p* < 0.05) or scrambled miRNA (*p* < 0.01)-treated neurons (Fig. 5*B*). In addition, we found that knockdown of CRIPT also led to a ∼20% decrease in the total size of the dendritic tree compared to GFP alone (*p* < 0.0002) or the scrambled miRNA (*p* < 0.05). To confirm the effect of CRIPT knockdown was in fact due to the reduced levels of CRIPT, we created an RNAi-resistant CRIPT cDNA (CRIPT^res^) by introducing silent mutations into the CRIPT miRNA target sequence (see Materials and Methods). We confirmed that CRIPT^res^ was resistant to knockdown by the CRIPT miRNA, compared to the WT version of CRIPT (CRIPT^wt^) when expressed in HEK293 cells (Fig. 5*C*). With this tool at our disposal, we compared the dendritic architecture of 3 experimental groups: (1) GFP + scrambled miRNA, (2) GFP + CRIPT miRNA, and (3) GFP+ CRIPT miRNA + CRIPT^res^. Group differences were seen in branch number (*H*_(2,73)_ = 9.846, *p* = 0.007) and total dendrite size (*H*_(2,73)_ = 9.364, *p* = 0.009). CRIPT knockdown again reduced the total number of branches (*p* < 0.05), as well as the total size (*p* < 0.01), of the dendritic tree compared to the scrambled miRNA (Fig. 5*D*). Rescuing CRIPT expression in CRIPT knockdown neurons using the CRIPT^res^ cDNA completely reversed this effect. There was no difference in any of the dendritic parameters tested compared to the scrambled miRNA (Fig. 5*D*). These data suggest that CRIPT is required for proper dendritic growth *in vitro* in mammalian spinal cord neurons.

### CRIPT is not sufficient to induce growth of the dendritic tree

We next asked whether increasing CRIPT expression influenced dendrite architecture. At the outset, we considered the possibility that only a limited number of CRIPT-binding sites exist at the plasma membrane. This would potentially mask a dendrite growth promoting phenotype of CRIPT overexpression. To test this idea, we overexpressed two different versions of CRIPT in spinal cord neurons: (1) WT CRIPT and (2) a version of CRIPT that was palmitoylated at the N terminus of CRIPT to allow CRIPT to traffic to the cell surface (pal-myc-CRIPT). The palmitoylation sequence was derived from paralemmin-1 and placed on the N terminus of CRIPT. Subcellular fractionation followed by Western blotting confirmed that palmitoylated CRIPT (pal-myc-CRIPT) is found predominantly in the membrane fraction, whereas CRIPT without the palmitoylation sequence is predominantly cytosolic in this preparation (Fig. 5*E*). To determine whether overexpression of these CRIPT constructs influenced dendritic architecture, we compared 3 groups: (1) +GFP, (2) +WT CRIPT, and (3) +pal-myc-CRIPT. By Kruskal-Wallis *H* test, no group differences were found (Fig. 5*F*). Taken together, these overexpression studies indicate that CRIPT alone is not sufficient to promote dendrite growth on its own, even when CRIPT is artificially targeted to the plasma membrane to overcome any potential saturation of CRIPT membrane-binding sites.

### CRIPT is required for the dendrite growth promoting activity of SAP97

**Figure 6.**
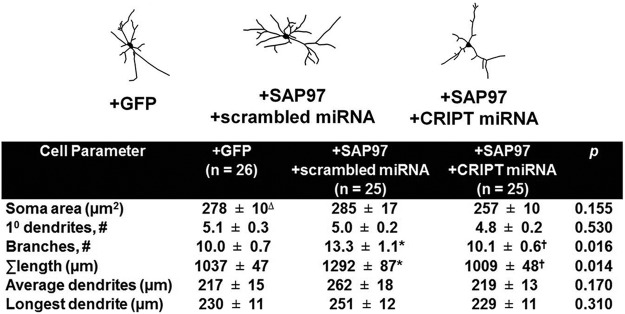
CRIPT functions downstream of SAP97 in promoting dendrite growth. Mixed spinal cord cultures were used for morphometry. Knockdown of CRIPT prevents growth and branching caused by SAP97 overexpression in mixed spinal cord neurons; *differs from +GFP group, *p* < 0.05; †differs from +SAP97 + +scrambled miRNA group, *p* < 0.05.

Prior work has shown that SAP97 promotes dendritic growth of motor neurons *in vitro* and *in vivo* (Zhang et al., 2008; Zhou et al., 2008). We next asked whether CRIPT was required for the dendritic growth promoting activity of SAP97. To address this question, three groups of neurons were studied: (1) +GFP, (2) +SAP97 + scrambled miRNA, and (3) SAP97 + CRIPT miRNA. Group differences were seen in branch number (*H*_(2,73)_ = 8.212, *p* = 0.016) and total dendrite size (*H*_(2,73)_ = 8.592, *p* = 0.014) . In the presence of the scrambled miRNA, overexpression of SAP97 led to a ∼30% increase in the total length and ∼30% increase in branch number of the dendritic tree compared to transfection with GFP alone (*p* < 0.05; Fig. 6) consistent with past findings (Zhang et al., 2008). The promotion of dendritic growth by overexpression of SAP97 was entirely blocked when CRIPT was knocked down in the same group of neurons (Fig. 6). These observations suggest that CRIPT is required for the dendrite growth-promoting actions of SAP97 and is part of the machinery required for SAP97-dependent developmental phenotypes.

### Loss of CRIPT influences the expression of select components of excitatory synapses

**Figure 7.**
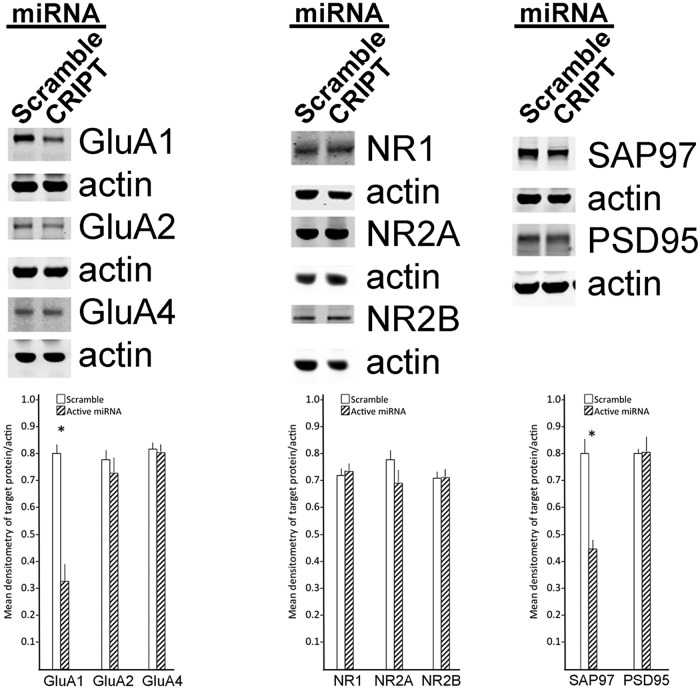
CRIPT knockdown leads to a selective reduction in the abundance of GluA1 and SAP97. Mixed spinal cord cultures were infected with HSV engineered to express a miRNA targeting CRIPT or a scrambled control. Two days later, lysates were prepared and subjected to Western blottings. No more than six independent experiments were performed for the quantitative image analysis. CRIPT knockdown leads to a reduction in GluA1 and SAP97 abundance and no effect on the abundance of GluA2, GluA4, NR1, NR2A, NR2B, or PSD95. Representative images of Western blottings with actin loading controls are shown and quantification of band intensity in the bar graphs below; *significant difference between groups, *p* < 0.05.

Passafaro et al., reported that CRIPT influences postsynaptic MAGUK clustering without affecting NMDA-Rs or synaptophysin-labeled presynaptic elements (Passafaro et al., 1999). These observations and conclusions were based on use of a cell-penetrating peptide (16 amino acids of the antennapedia homeodomain) linked to the C-terminal nine amino acids of CRIPT (“antp-CRIPT”). While antp-CRIPT had the virtue of competing (and therefore blocking) the interaction all proteins that bind to the C terminus of CRIPT, this reagent also acts as a dominant-negative inhibitor of ligands for PDZ3 of all MAGUKs. Thus, the specific role of CRIPT in synaptic molecular biology is undefined. To begin to address this issue, we knocked down CRIPT in spinal cord cultures using the validated miRNA described above and prepared lysates for Western blottings. We find that knockdown of CRIPT led to a significant reduction in the abundance of GluA1 and SAP97, but had no effect on the abundance of GluA2, GluA4, NR1, NR2A, NR2B, or PSD95 (Fig. 7). Thus, loss of CRIPT leads to very specific changes in components of AMPA-Rs and PSD MAGUKs. These observations are the first clues suggesting that CRIPT can regulate excitatory neurotransmission (through changes in heterooligomeric assembly of AMPA-Rs) and downstream consequences (through changes in the composition of the PSD). The use of dynamic reporters of synaptic function, electrophysiology and super-resolution microscopy will be required in the future to precisely define the molecular mechanisms of CRIPT action on synaptic biology.

### CRIPT promotes dendritic growth in vivo

Our understanding of *in vivo* biology of CRIPT is hampered by the fact that CRIPT null mice die at embryonic day 9 and no conditional alleles exist. One potential path forward is to study the *in vivo* effects of CRIPT in phylogenetically lower animals. CRIPT is an ancient gene and was likely to exist in the last common ancestor before the split of the Animalia and Plantae kingdoms of life. The *C. elegans* genome contains a CRIPT ortholog (C36B1.14) that is 38.8% identical to hCRIPT and both proteins are very basic (pI hCRIPT is 9.57 and pI of *C. elegans* CRIPT is 9.61). CRIPT message is expressed in embryonic (Moore et al., 2005; Stetina Von et al., 2007), early larval (Stetina Von et al., 2007), and adult (Kaletsky et al., 2016) neurons (http://www.vanderbilt.edu/wormdoc/wormmap/WormViz.html). The cardinal feature of hCRIPT is 8 cysteine residues and *C. elegans* CRIPT has seven cysteine residues in register with their locations in the human protein. A mutant allele of *cript* allele exists (*tm430* contains ∼400-bp deletions near the C-terminal region of the protein, which would eliminate CRIPT’s PDZ-interaction motif) and is likely to be a null. In light of these considerations, we studied of the *in vivo* biology of CRIPT biology in *C. elegans.*

**Figure 8.**
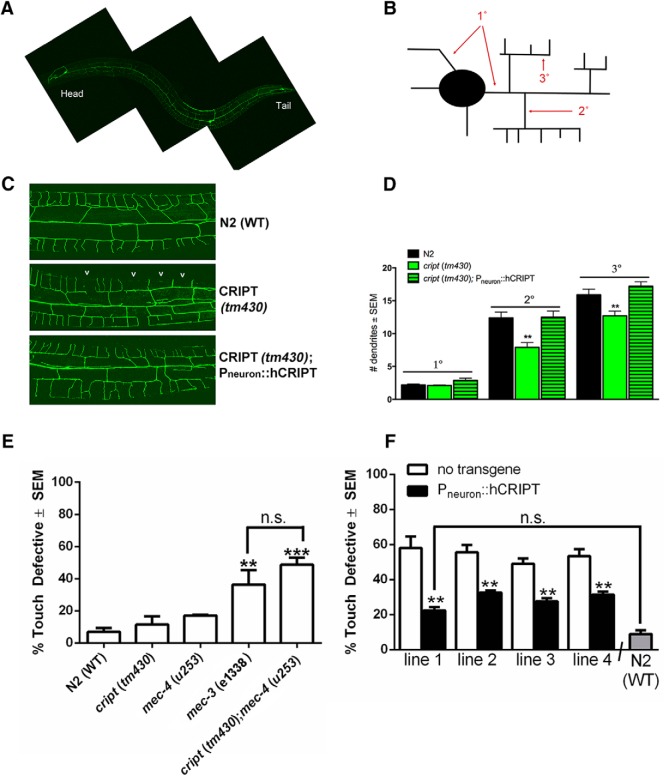
Loss of *cript* (*tm430*) decreases dendrite branch number *in vivo* and results in a mechanosensory defect; rescue with hCRIPT. ***A***, Representative full body image of PVD neuron labeled with GFP in the WT (N2 Bristol strain) young adult (L4 stage + 1 d old). ***B***, Schematic of PVD neuron and its primary (1˚), secondary (2˚), and tertiary (3˚) branches used for quantification. ***C***, Representative images of the PVD neuron labeled with GFP of indicated genotypes in the tail region used for quantification at the young adult stage (L4 stage + 1 d old). Gaps in the dendritic tree of *cript* (*tm430*) worms are noted with “v”. ***D***, Quantification of the number of primary (1˚), secondary (2˚), and tertiary (3˚) branches in indicated strains (*n* ≥ 10 per genotype). Analyst was blinded to genotype during quantification. We found that *cript* (*tm430*) mutants have a significant decrease in secondary (**p < 0.01; *post hoc* test following one-way ANOVA; *F*_(3,37)_ = 6.781; *p* = 0.0009) and tertiary (**p < 0.01; *post hoc* test following one-way ANOVA; *F*_(3,37)_ = 6.365; *p* = 0.0014) branch number. This was completely rescued by overexpression of the human ortholog of CRIPT in the nervous system using the unc-119 promoter (P_neuron_), which also increased the number of primary dendrites compared to WT animals (*p < 0.05); *post hoc* test following one-way ANOVA; *F*_(3,37)_ = 3.569; *p* = 0.0232). ***E***, WT, *cript* (*tm430*), *mec-3 (e1338)*, and *cript* (*tm430*); *mec-4* (*tu253*) animals were scored for their response to touch. Average of three independent experiments is shown. Experiments were all completed with analyst blinded to genotype. We found that *cript* (*tm430*); *mec-4* (*tu253*) animals show a synthetic touch defect (**p <0.01 according to a *post hoc* test following a one-way ANOVA). We also confirmed that mec-3 (CB1338) animals are touch defective compared to WT counterparts (***p < 0.005 according to a *post hoc* test following a one-way ANOVA; *F*_(4,10)_ = 11.93; *p* = 0.0008). ***F***, Touch defect is suppressed in *cript* (*tm430*); *mec-4* (*tu253*) animals by overexpression of the human ortholog of CRIPT in the nervous system using the unc-119 promoter (P_neuron_::hCRIPT) in four independent extrachromasomal array lines (**p <0.01 according to a *post hoc* test following a one-way ANOVA when compared to nontransgenic counterparts). One strain (line 1) fully rescued the touch defect when compared to WT in a *post hoc* test following a one-way ANOVA (*F*_(8,18)_ = 26.45; *p* < 0.0001).

We began by asking whether loss of CRIPT influences the size and complexity of the dendritic tree of a single neuron. To this end, we used a *C. elegans* reporter strain with GFP expressed in the well-characterized PVD mechanosensory neuron (Smith et al., 2010; Smith et al., 2013). The PVD neuron dendrites showed an ∼25% decrease in the branches of secondary and tertiary dendrites in the *cript(tm430)* background versus the WT (N2 Bristol strain) background (*p < 0.05; Fig. 8*A-D*). We next asked whether the dendrite growth defect of the *cript* mutant was due to the loss of expression of CRIPT in neurons. To study this, we expressed hCRIPT under the control of a nervous system specific promoter (P_unc-119_::hCRIPT) (‘P_neuron_::hCRIPT’) in *cript* (*tm430*) mutants with GFP labeled PVD neurons. We found that neuronal expression of hCRIPT was sufficient to rescue the decrease in secondary and tertiary branches in the *cript* (*tm430*) mutant to WT levels (Fig. 8*D*). This effect was seen in three independently generated transgenic lines (data not shown). Although we have no direct evidence that CRIPT is expressed at physiologically relevant levels in PVD neurons, the fact that pan-neuronal expression of hCRIPT rescues the abnormalities in the PVD neurons dendrites in the *cript(tm430)* background, enables two observations: (1) the loss of neuronal CRIPT is responsible for the dendrite phenotype of PVD neurons in the *cript(tm430)* animals, and (2) while parsimonious to infer that changes in CRIPT within PVD neurons themselves account for these observations, our results are equally consistent with the idea that CRIPT has non cell-autonomous effects on neuronal biology in the worm. In this later scenario, CRIPT expression in non-PVD neurons might influence network activity, for example, and thereby influence PVD neurons. It is also worth remembering that PVD neurons are sensory and the extent to which they are activated by glutamate receptors (as with our studies of mammalian neurons *in vitro*) is an open question. Regardless of these specific details, our observations are the first *in vivo* demonstration that CRIPT is essential for normal dendrite elaboration and illustrate the remarkable evolutionary conservation CRIPT function.

### Loss of cript causes a mechanosensory defect in vivo

We wondered whether the anatomic defects in PVD neuron dendrites incurred by loss of *cript* had functional consequences. *C. elegans* response to gentle stroking by an eyelash is mediated by six neurons: ALML/R (where “L” and “R” refer to the left and right side of the body), PLML/R, AVM and PVM (Chalfie and Sulston, 1981). This “light touch” response is lost in animals with mutation in the amiloride-sensitive sodium channel encoded by the *mec-4* gene. However, animals with mutation in *mec-4* do respond to a stronger mechanical stimulus (e.g., touching the midsection of the animal with a platinum wire) and this response is lost when the PVD neuron is eliminated by laser ablation (Way and Chalfie, 1989). The “harsh touch” response is mediated by ALML/R, PLML/R, PVD, and perhaps the FLP sensory neurons. PVD function is typically assayed in a *mec-4* mutant background as this eliminates the light touch response and thus isolates PVD-dependent behaviors (Liu and Shen, 2011). As a positive control in touch assays, investigators use *me*c-3(*e1338*) animals because they are insensitive to both light and harsh touch (Way and Chalfie, 1989; Liu and Shen, 2011). We took advantage of this simple yet powerful system to inquire if the reduced branching of the dendritic arbors of the PVD neuron seen in *cript* mutants was associated with a functional deficit in the harsh touch assay. We compared the response to harsh touch in five *C. elegans* strains: (1) the WT N2 (a negative control), (2) *mec-3(e1338)*, (3) *cript* (*tm430*), (4) *mec-4*(*u253*), and (5) *cript* (*tm430*); *mec-4* (*u253*) double mutants (Fig. 8*E*). By ANOVA, group differences were found (*F*_(4,10)_ = 11.93; *p* = 0.0008). Using *post hoc* analysis, we confirmed that ∼40% of *mec-3*(*e1338*) mutant animals were defective to harsh touch (*p* < 0.05; they did not respond or move in response to stimulus) in comparison to WT animals, in which only 10% displayed a lack of response to harsh touch. The *cript* (*tm430*);*mec-4* (*u253*) double animals displayed a significant (*p* < 0.01) defect to harsh touch in ∼50% of animals compared to WT animals. There were no significant differences between the single mutants [i.e., *cript* (*tm430*) or *mec-4* (*u253*)] and N2 worms (Fig. 8*E*). These observations indicate that the *cript* mutants are incapable of compensating for the loss of *mec-4*. This functional impairment could be due to the observed abnormality in dendrite growth of *cript* mutants.

Finally, we asked whether expression of hCRIPT in the nervous system of *cript* mutant (*tm430*) worm would restore harsh touch sensitivity in the *cript* (*tm430*);*mec-4* (*u253*) animals. We found that expression of hCRIPT in the nervous system (P_neuron_::hCRIPT) was sufficient to partially rescue the harsh touch defect of the *cript* (*tm430*);*mec-4* (*u253*) animals. Approximately ∼55% percentage of *cript* (*tm430*);*mec-4* (*u253*) animals showed a harsh touch defect and this was reduced to ∼25% when hCRIPT was expressed in the nervous system (*p* < 0.01). This effect was found in 4 independently generated transgenic lines (Fig. 8*F*). These observations suggest that neuronal CRIPT is required for normal touch responses in *mec-4*(*u253*) mutant animals. Although pan neuronal expression of hCRIPT both rescues the PVD neuron dendrite abnormality and touch sensitivity *cript* (*tm430*); *mec-4* (*u253*) animals, our data do not distinguish between a cell autonomous versus cell nonautonomous role of CRIPT. It remains possible that CRIPT expression in neurons, in addition to the touch critical neurons such as PVD underlie, these observations.

## Discussion

hSAP97 is a modular scaffolding protein that localizes to the PSD and plays an essential role in translating the activity of AMPA-R assembled with GluA1 into appropriate dendrite growth and ensuring proper circuit patterning (Zhang et al., 2008; 2015). We show here that CRIPT is a *bona fide* binding partner of the prodendrite growth domain of SAP97, PDZ3. Our observations provide novel insights into the currently limited knowledge about CRIPT biology by showing that CRIPT is required for normal dendrite growth both *in vitro* and *in vivo*. In addition, we show that the loss of CRIPT in neurons causes behavioral defects, highlighting the functional importance of CRIPT in organizing neuronal circuits. The relevance of our results is suggested by the identification of children with mutations in CRIPT. Two children with homozygous, likely null, mutations are microcephalic and severely encephalopathic (Shaheen et al., 2014); another child with a compound heterozygous mutation is also microcephalic and displays global developmental delay (Leduc et al., 2016) Thus, while MAGUKs such as PSD95 and SAP97 can substitute for each other in electrophysiological assays (Schlüter et al., 2006; Howard et al., 2010), our data indicate that CRIPT has a nonredundant and critical cell biological function in neurons.

Many factors influence the computational work of neurons including: (1) the size, geometry, and complexity of the dendritic tree (Stuart et al., 1999); (2) the quantitative and qualitative nature of the afferent input (Hume and Purves, 1981; Inglis et al., 2002); (3) the molecular composition of synapses including which receptor-channels reside in the synapse and their dynamic entry into and egress from synapses; and (4) the molecular composition of the PSD. The hundreds of proteins in the PSD are the molecular actors that translate synaptic activity into lasting changes in synaptic function. Synaptic activity can play a critical role in synaptic dynamics and one form of activity-dependent development involves activation of the NMDA subtype of glutamate receptor (Cline et al., 1987; Kleinschmidt et al., 1987; Kalb, 1994). NMDA-R-mediated dendrite growth and synaptic specification involves: (1) elaboration of extracellular factors, such as BDNF, Wnts, and nitric oxide (Wu et al., 1994; McAllister et al., 1995; Cramer et al., 1996; Inglis et al., 1998; Ha and Redmond, 2008); (2) activation of intracellular signaling molecules (Wu and Cline, 1998; Li et al., 2000; Li et al., 2002; Redmond et al., 2002; Sin et al., 2002; Yu and Malenka, 2003; Gaudillière et al., 2004; Wayman et al., 2006; Ha and Redmond, 2008; Peng et al., 2009); and (3) new gene expression (Redmond et al., 2002; Gaudillière et al., 2004; Shalizi et al., 2006; Li et al., 2009). CRIPT directly and indirectly interacts with multiple components of the PSD and is thus well positioned to modulate neuronal computations.

### CRIPT interactome

Sequence analysis of CRIPT reveals domain architectures such as zinc-fingers, tetratricopeptide-like helical domains, JmjC domains, and aconitase/3-isopropylmalate dehydratase, swivel domains (see InterPro, EMBL-EBI). Protein interaction databases provide evidence for CRIPT binding to all at least 11 proteins (see IntAct database, EMBL-EBI). These *in silico* and unbiased large-scale screening studies suggest that CRIPT interacts with a wide variety of proteins and may be able to bind DNA and/or double-stranded RNA. Our work shows that CRIPT can be localized to synapses, as well as the cell body, of neurons, suggesting CRIPT has important functions in several sites within the cell.

CRIPT binds PDZ3 of synaptic MAGUKs and this interaction is required for association of PSD95 with microtubules (discussed further below; Passafaro et al., 1999). Other biological actions of CRIPT, such as synaptic clustering of MAGUKs (e.g., PSD95, PSD93 and GKAP; Passafaro et al., 1999) and dendrite growth (Fig. 5) may also depend on this interaction but this is not known. It is worth noting that both *C. elegans* and *D. melanogaster* CRIPT orthologues have a C-terminal threonine rather than valine (as in mammals). While these two amino acids are sterically similar, there are no known examples of a native PDZ ligand ending in threonine. If exchanging threonine with valine weakens the interaction of worm CRIPT with a PDZ domain-containing protein, it suggests that the biological actions of CRIPT on dendrites could be at least partially independent of a CRIPT/PDZ domain interaction. Future studies will be required to understand which protein-protein interactions subserve CRIPT effects on neurons.

Prior work suggests that CRIPT binds microtubules (causing them to bundle) and facilitates recruitment of MAGUKs to bundled microtubules in a heterologous expression system (Niethammer et al., 1998; Passafaro et al., 1999). Since the publication of these studies almost 20 years ago a more nuanced view of microtubule-binding proteins has emerged and this raises questions about the original observations regarding CRIPT and microtubules. First, whether recombinant CRIPT directly binds purified microtubules has not been demonstrated and so some of the original observations may be due to indirect effects. Second, some proteins that associate with the cytoskeleton, when overexpressed, can decorate microtubules indiscriminately and reorganize with the cellular microtubule network in nonphysiologic ways. How endogenous CRIPT interacts with microtubules and controls their behavior is unclear. Third, immunogold electron microscopic studies show that CRIPT concentrates in the PSD along with MAGUKs (Niethammer et al., 1998), and this is consistent with our own immunocytochemical localization studies (Fig. 3). On the other hand, large stable microtubules decorated with microtubule associated protein 2 reside in dendritic shafts and are absent from spines (Landis and Reese, 1983; Matus, 2000; Kaech et al., 2001). Even if endogenous CRIPT bound microtubules, the extent to which this occurs at synapses is unknown. We believe that while a stable MAGUK-CRIPT-microtubule complex is unlikely to exist at synapses, these observations do not exclude an ephemeral complex. Dynamic microtubules decorated with plus-end tracking proteins actively explore spines (Gu et al., 2008; Hu et al., 2008; Jaworski et al., 2009; Merriam et al., 2011; 2013). It has been estimated that roughly 1% of spines contain polymerized tubulin at any one time and that every spine will have been invaded by microtubules every 24-h period (Hu et al., 2008). If CRIPT is truly a microtubule-binding protein, it could transiently link invading microtubules to the PSD.

### CRIPT cell biology

This work raises a number of issues for future investigation. First, on the basis of the direct physical interaction of CRIPT with SAP97 and other PSD MAGUKs, does CRIPT influence MAGUK-mediated process such as: (1) clustering and stabilizing glutamate receptors (Chen and Featherstone, 2005; Elias et al., 2006; Waites et al., 2009), (2) regulating spine size and density (Nikonenko et al., 2008), and (3) trafficking of glutamate receptors to the cell surface (Nicoll et al., 2006; Elias and Nicoll, 2007). Second, several aspects of synaptic biology are influenced by activity and whether CRIPT plays a role in these dynamic processes is an open question. Third, dendritic distribution of CRIPT only partially overlaps with MAGUKs and it is present at some but not all synapses. More advanced imaging technologies may help inform us about the spatiotemporal relationships between CRIPT, synapses, and its myriad binding partners. Fourth, do familial mutations in CRIPT impact dendritic and synaptic biology?

Understanding how activity-dependent development of the dendritic tree is promoted may help develop tools that can promote recovery following injury to the central nervous system. For instance, repetitive activation of motor circuits in experimental animals or humans has been shown to improve rehabilitation in patients following motor injury (Dietz et al., 1995; Wernig et al., 1995; Edgerton et al., 2001; Gazula et al., 2004; Ivey et al., 2008; Marchal-Crespo and Reinkensmeyer, 2009; Lo et al., 2010). This is consistent with our findings that activity promotes dendritic remodeling that in turn can promote development of normal locomotor behavior (Inglis, et al., 2002; Zhang, et al., 2008; Zhou, et al., 2008).

## Synthesis

Reviewing Editor: Christophe Bernard, INSERM & Institut de Neurosciences des Systèmes Decisions are customarily a result of the Reviewing Editor and the peer reviewers coming together and discussing their recommendations until a consensus is reached. When revisions are invited, a fact-based synthesis statement explaining their decision and outlining what is needed to prepare a revision will be listed below. The following reviewer(s) agreed to reveal their identity: Ulrich Thomas, Michael Francis.

Synthesis

There was a general consensus in the reviews. Demonstrating cell-specific rescue in PVD is a very simple experiment for a C. elegans lab (PVD-specific promoters are available and actually use used in the paper they cite), but perhaps not simple for a lab that does not have expertise in this area. As an alternative, we suggest that the authors could simply show evidence for expression in PVD. This could be accomplished with either a transcriptional reporter using the promoter or even by examining available expression information on Wormbase. Hence, although more experiments are not strictly required, adding some would reinforce the message. At the very least, limitations should be discussed, as detailed below.

1. Some results appear stated too strongly or are over-interpreted. For example, the imaging studies in Figs 3 and 4 appear to show that CRIPT is evenly distributed throughout the neuron. The studies of colocalization with synaptic markers are therefore difficult interpret. Some percentage of the signal overlaps because CRIPT signal is almost ubiquitous throughout the labeled neurons. This seems likely to be due at least in part to the way the experiment was performed (RNAi of endogenous CRIPT in combination with expression of HA-CRIPT). The authors present a logical rationale for choosing this strategy, but they should more directly acknowledge the potential limitations of the approach, and limit descriptive discussions of dendritic CRIPT structures and synaptic localization, as the images presented don't provide compelling support for subcellular localization to specific structures.

Fig 1H: the quality of this WB is somewhat limited. If possible, provide a version without simultaneous staining for both proteins or add at least molecular weight markers and comment on the additional bands in the input lane (spill over from Ig control lane ?) .

The Kd for the PDZ- CRIPT interaction as determined by surface plasmon resonance was 6.57 �M - how does this value compare to other canonical PDZ interactions ?

2. The paper should be reorganized to improve its focus.

-The structural modeling (Fig 2), as presented, does not add to the paper.

-The negative date for CRIPT OE (Fig. 6) should be consolidated with Fig. 5 and integrated into that section of the paper.

-I would suggest changing the order of the figures, moving forward the C. elegans data suggesting a potentially conserved function and ending with the data showing that CRIPT downregulation alters GluA1 and SAP97 levels (current Fig. 8). This would provide an opportunity to discuss potential mechanism of action.

3. The analysis of C. elegans CRIPT, while interesting, needs to be strengthened and corrected. Specifically, data showing a requirement for CRIPT in PVD (not simply pan-neuronal expression) or data showing that CRIPT is normally expressed in PVD is required to strengthen the analysis.

In contrast to mammalian CRIPT the orthologue in C.elegans (also in fly) does not terminate in valine but rather in threonine. Still the rescue experiment suggests conservation of CRIPT function. While Thr and Val are sterically similar, I am not aware of ligands with terminal Thr to bind to PDZ domains - are there examples ? What other residues that are involved in the binding model (Figure 2) are conserved. Please discuss.

4. Dendritic tree morphology:

I am not that familiar with details in regard to the used model systems. I therefore wonder, whether the knock-down of CRIPT after infection would be expected to have more severe effects if applied earlier (also considering that there is some delay in the downregulation of CRIPT protein after shRNA and that knockdown is probably not 100%) - in the C.elegans model there is a consitutive knock-out and it leads to defects to a degree similar to that observed in the shRNA experiments (i.e. it does not seem to be much more severe). Can you elaborate on this aspect in the discussion ? Also I miss a bit more about other factors that might be involved in the observed phenotypes (I guess there are many mutations that cause similar phenotypes- are there for instance some that might be considered as potential downstream effectors of SAP97 ?) In other words I would like the authors to discuss their findings in a somewhat broader cell biological context.

-Methods for the touch assay should be provided. The text describes the stimulus used as “light touch”. However, assays for PVD function typically involve harsh touch at the level of the vulva as described in the Way & Chalfie paper that the authors reference.

-Discussion of the results in worms should acknowledge that the effects are unlikely to involve the same mechanism as is implied for spinal cord neurons. PVD neurons are sensory and therefore dendrite elaboration is unlikely to involve synaptic activation of glutamate receptors.

-The statement “C. elegans utilize two neurons, MEC-4 and PVD, to sense and respond to touch” is incorrect. C. elegans utilize 6 neurons (ALML, ALMR, AVM, PVM, PLML, PLMR) to respond to light touch along the body wall and 6 additional neurons (2 ADE, 2 PDE, 2 PVD), to respond to harsh touch along the body wall. mec-4 is not a neuron, but a gene expressed by mechanosensory neurons and required for their function.

-The description of the mec-4 allele employed appears incorrect. I believe the authors are referring to u253 rather than tu253.

-Figure 3 (and respective text/legend): red labeling should be “ HA-CRIPT” rather than just CRIPT.

-Figure 4: as for Figure 3 it is not evident from labelings, the legend or main text that HA-CRIPT and (I guess) EGFP-SAP97 are monitored here. This can only be deduced from the methods section.

-CRIPT seems to be widely expressed in dendrites and somata - how about axons ? The description of its subcellular distribution is, let's say, “unconventional”, e.g. “...elongated cylinders, some reaching 5 �m in length...”. Without appropriate counterstainings for markers that could help defining this pattern of staining, I would recommend shortening of this description and being very careful about using the term “co-localization” (e.g. true co-localization of synaptophysin and CRIPT would indicate presynaptic localization of the latter). I would also suggest to bundle Figures 3 and 4 into one figure, maybe on the expense of some of the overview panels.

-The interpretation “ We propose that at least some excitatory synapses.... ... fundamental relationship between these proteins” would better fit into the discussion. (Similarly for the statement further downwards by the end of the paragraph mentioning possible physiologial effects due to reduction of CRIPT).

Also, in the discussion the possible interaction between MTs and CRIPT is reviewed with scepticism, which is okay on one hand, but on the other hand the evidence that CRIPT exerts its effects primarily via its PSD entity (and thus via GluA1) appears also somewhat weak to me, as for instance spine localization is not evident from the images. Couldn't transport of GluA1 along MTs aIso play a role that involves CRIPT? I would therefore suggest to balance this argumentation accordingly.

-Functional interaction of SAP97 and CRIPT:

The promoting effect of SAP97 overexpression on dendritic complexitiy is reverted by CRIPT RNAi. This is consistent with CRIPT being required for SAP97 stability. Overexpressing of CRIPT had no promoting effect of dendritic complexity, suggesting that SAP97 is limiting at this point. Have you tried co-overexpression in order to evaluate the possibility of synergistic effects ? Do the findings rule out that CRIPT has an GluA1- and/or SAP97-independent role in denditrogenesis ?

-You cite your own publications for the interaction between SAP97 and GluA1 (“In light of the known physical interaction between GluA1 and SAP97 (Zhou et al., 2008; Zhang et al., 2015)”) - but wasn't this interaction shown already before ?

-“...some of which appear to be spines or filopodia”... “In our spinal cord neuron cultures we do not typically see dendritic spines (Zhang et al., 2015)” - this is a bit confusing.

-Some phrasings where I (as a non-native speaker) feel that grammar might not be correct , for instance(?):

-The binding of the C-terminal three residues of CRIPT to PDZ3 of PSD95 and SAP97 are exactly the same.

...none of the commercially available antibodies to CRIPT were suitable...

Figure 3. CRIPT partially co-localizes synaptophysin containing synapses

albeit all lower levels
